# The Role of Animal-Assisted Therapy in Enhancing Patients’ Well-Being: Systematic Study of the Qualitative and Quantitative Evidence

**DOI:** 10.2196/51787

**Published:** 2024-03-18

**Authors:** Ramendra Pati Pandey, Himanshu, Gunjan, Riya Mukherjee, Chung-Ming Chang

**Affiliations:** 1 School of Health Sciences & Technology, University of Petroleum and Energy Studies, Dehradun, Uttarakhand, India; 2 Graduate Institute of Biomedical Sciences, Chang Gung University, Taoyuan, Taiwan; 3 Master & PhD Program in Biotechnology Industry, Chang Gung University, Taoyuan, Taiwan; 4 Department of Medical Biotechnology and Laboratory Science, Chang Gung University, Taoyuan, Taiwan

**Keywords:** animal-assisted therapy, pet therapy, outcome assessment, policies, systematic study

## Abstract

**Background:**

Animal-assisted therapy, also known as pet therapy, is a therapeutic intervention that involves animals to enhance the well-being of individuals across various populations and settings.

**Objective:**

This systematic study aims to assess the outcomes of animal-assisted therapy interventions and explore the associated policies.

**Methods:**

A total of 16 papers published between 2015 and 2023 were selected for analysis. These papers were chosen based on their relevance to the research topic of animal-assisted therapy and their availability in scholarly databases. Thematic synthesis and meta-analysis were used to synthesize the qualitative and quantitative data extracted from the selected papers.

**Results:**

The analysis included 16 studies that met the inclusion criteria and were deemed to be of moderate or higher quality. Among these studies, 4 demonstrated positive results for therapeutic mediation and one for supportive mediation in psychiatric disorders. Additionally, all studies showed positive outcomes for depression and neurological disorders. Regarding stress and anxiety, 3 studies indicated supportive mediation, while 2 studies showed activating mediation.

**Conclusions:**

The overall assessment of animal-assisted therapy shows promise as an effective intervention in promoting well-being among diverse populations. Further research and the establishment of standardized outcome assessment measures and comprehensive policies are essential for advancing the field and maximizing the benefits of animal-assisted therapy.

## Introduction

The inclusion of animals in psychological treatment is not a recent or uncommon practice. Throughout history, there has been an understanding of the positive impact animals can have on human well-being [1]. This connection between humans and animals is deeply ingrained in our collective subconscious, influencing our emotional experiences [2]. The earliest documented instance dates back to the late 18th century when animals were introduced into mental health institutions to enhance social interaction among patients [3,4]. Today, numerous programs worldwide incorporate animals to varying degrees in their services. These programs are particularly beneficial for individuals who have experienced trauma, including those diagnosed with posttraumatic stress disorder (PTSD), schizophrenia, Alzheimer disease, autism, etc [4,5].

In the past 50 years, the field of human-animal interaction and, specifically, animal-assisted therapy (AAT) has made significant advancements and progress. AAT is a therapeutic approach that uses animals to improve overall health and well-being. It encompasses emotional, psychological, and physical interactions between individuals, animals, and the environment [6]. AAT interventions involve qualified treatment providers facilitating interactions between patients and animals with specific therapeutic goals in mind. These interventions often involve collaborative activities between human-animal teams, aiming to promote therapeutic and supportive outcomes [7]. AAT interventions contribute to individuals’ well-being, supporting physical health and improving cognitive, emotional-affective, and social aspects, leading to enhanced emotional well-being, reduced anxiety, and decreased stress levels [8-10].

Research on therapies involving human-animal interaction has focused on specific animals such as dogs, cats, or horses and specific populations such as those with autism [11]. Dogs, in particular, are commonly preferred for therapy due to their exceptional bond with humans in modern times. Over thousands of years of shared evolutionary history [1], dogs have acquired adept socialization skills with humans through processes of domestication and natural selection. They have become our loyal companions, developing unique social skills for interacting with humans [12]. For instance, studies indicate that dogs possess a sensitivity to our emotional states [13] and can interpret our social cues [14], even engaging in sophisticated communication through behaviors like gaze alternation [15]. Furthermore, dogs are capable of forming intricate attachment relationships with humans, resembling the bonds found in relationships between infants and caregivers [16]. Research suggests that among the various animals involved in AAT, dogs tend to exhibit superior interactions with people compared to other species, benefiting both children and adults [6].

This systematic review and meta-analysis sheds light on the potential of animal-assisted interventions to enhance overall well-being and health. Our research aims to contribute to the growing body of evidence supporting the use of animals in therapeutic contexts and to explore the specific contexts in which these interventions are most effective. One of the unique aspects of our study is the incorporation of both quantitative and qualitative analyses to provide a comprehensive understanding of the effects of AAT. While previous research has predominantly relied on quantitative data, we believe that qualitative insights from participants who have experienced these interventions offer valuable perspectives. The special bonds formed between humans and animals are recognized as essential catalysts for transformation and are held in high regard, similar to the therapist-client relationship.

## Methods

### Search Strategy

The meta-analysis was carried out following the methodologies outlined in the esteemed Cochrane Handbook for Systematic Reviews of Interventions [17], and the findings were reported in compliance with the PRISMA (Preferred Reporting Items for Systematic Reviews and Meta-Analyses) guidelines [18]. To ensure comprehensive coverage, electronic databases were meticulously searched up until June 2023. A total of five English-language electronic databases, including PubMed, Web of Science, Clinical Trials, Science Direct, and Google Scholar, were meticulously explored. This thorough exploration entailed using a combination of pertinent controlled vocabulary terms (eg, Medical Subject Headings [MeSH]) and relevant free-text terms. The search strategy used can be summarized as follows: (animal assisted therapy OR animal assisted intervention OR animal assisted activity OR animal activity interaction OR animal assisted method OR animal facilitated therapy OR pet therapy OR canine assisted therapy OR dog assisted therapy) AND (quasi-experimental study OR randomized controlled trial) AND (pain OR anxiety OR depression OR blood pressure OR BP OR heart rate OR HR) AND (work-related stress OR workplace health OR employee well-being OR burnout) AND (tumor OR malignant OR carcinoma OR oncology OR hospitalization OR hospitalized patients OR inpatients). By using this extensive and refined approach, the meta-analysis aimed to capture a comprehensive body of evidence on the effects of AAI on various health outcomes.

### Inclusion and Exclusion Criteria

The inclusion criteria were set based on the PICOS (Patient, Problem, or Population; Intervention; Comparison; Outcomes; and Study Design) framework: studies evaluating the effects of AAT, animal-assisted intervention, or animal-assisted activity; studies evaluating the effects of animal interactions on health and well-being (including depression, agitation, loneliness, stress, and quality of life), social interaction, engagement, physical function, behavioral symptoms, medication use, and adverse events; articles published in English; studies available in full-text format; and studies using quasi-experimental designs or randomized controlled trials. To maintain the rigor and relevance of the study, publications that lacked sufficient information regarding the therapy or did not involve an animal intervention were excluded from consideration.

### Data Synthesis

In our study, we used thematic synthesis as a method to assess the eligibility and quality of the articles [19]. Each article was independently reviewed to determine its suitability for inclusion. We followed a traditional methodology for evaluating the papers that involved examining factors such as the presence of adequate control groups, control of confounders, randomization, well-described experimental design, and relevant outcome variables. Articles that met these criteria were selected and organized into a single sheet using Microsoft Office Excel (2019; Microsoft Corporation). For the included studies, we extracted and compiled various data points into a structured table. This information encompassed the author’s name, country of publication, year of publication, patient characteristics (including sample size, age, gender, and target group), type of study, study design, description of AAT, type of intervention, control group details, study duration, outcomes measured, and the authors’ conclusions. To effectively manage the papers, we used Mendeley software (version 1.19.8; Elsevier).

### Classification

To determine the specific contexts in which AATs are effective, we classified the interventions into three categories. First, “supportive mediation” involves AATs providing emotional and psychological support to individuals. Second, “therapeutic mediation” entails AATs addressing specific therapeutic goals and needs in a structured manner. Finally, “activating mediation” comprises AATs designed to stimulate engagement and participation in various activities or tasks.

### Risk of Bias

Assessing publication bias is a crucial component in safeguarding the strength and credibility of our meta-analysis, which examines the effects of AAT on improving the well-being of individuals in diverse populations and settings. To gauge the possible influence of publication bias on our results, we applied several established techniques recommended in the field. One of these methods involved visually examining a bias risk graph for signs of asymmetry, which can be an indication of publication bias. By using these comprehensive approaches, our objective was to address any potential bias and guarantee that our meta-analysis offers an impartial synthesis of the existing evidence regarding the beneficial effects of AAT.

## Results

### Study Selection

**Figure 1.**
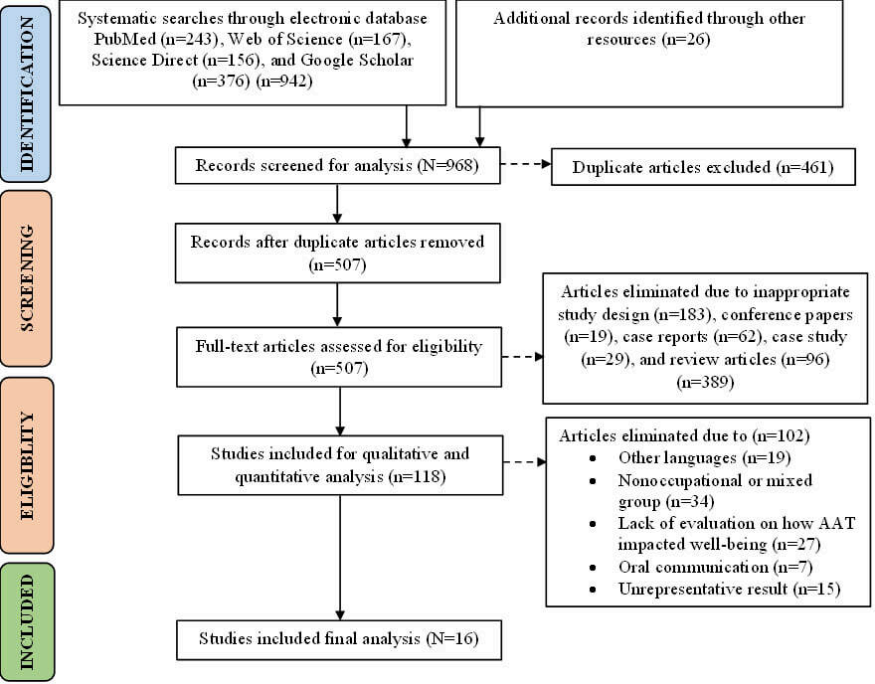
Literature screening flowchart (PRISMA; Preferred Reporting Items for Systematic Reviews and Meta-Analyses). AAT: animal-assisted therapy.

The outcome of the search is depicted in Figure 1. The search process resulted in 968 unique articles after initial searches from various electronic databases like PubMed, Web of Science, Science Direct, and Google Scholar, which yielded 942 articles. An additional 26 articles were extracted from other sources. After eliminating duplicate articles, the total number of articles was reduced to 507. Subsequently, the articles were assessed based on their title and abstract to determine eligibility. Among the initial pool, 389 articles were excluded as they did not meet the eligibility criteria, mainly due to the lack of relevance to AAT. After reading the full text of the remaining articles, 102 more articles were excluded. Of these 102 articles, 60 did not meet the inclusion criteria, and 42 were excluded due to being classified as nonoccupational mixed groups or having unrepresentative results. Finally, a total of 16 studies that met the inclusion criteria were included in the final analysis. The findings and details of these 16 studies are summarized in Tables1  2.

**Table 1.** Summary of outcomes from studies included.

Author; year	Type of disorder	Mediation	Study outcome of AAT^a^	Conclusion
Shih and Yang [20]; 2023	Psychiatric disorder	Therapeutic	Mental Health–Social Functioning Scale, Social Adaptive Function Scale, Taiwanese version of the World Health Organization Quality of Life	Social functioning was significantly higher in the experimental group; quality of life improved
Chen et al [21]; 2022	Psychiatric disorder	Therapeutic	Montreal Cognitive Assessment, chair stand test, Timed Up and Go, 5-min walk test, Assessment of Communication and Interaction Skills	Significant improvements in communication and interpersonal skills; improved lower extremity strength and social functions
Allen et al [22]; 2022	Stress and anxiety	Activating	Service Satisfaction Scale, Posttraumatic Stress Disorder Reaction Index for DSM-5, Strengths and Difficulties Questionnaire, Moods and Feelings Questionnaire, Screen for Child Anxiety Related Disorders	Improvements in caregiver-reported PTSD^b^ symptoms, internalizing concerns, and externalizing problems
Chen et al [23]; 2021	Psychiatric disorder	Therapeutic	Positive and Negative Syndrome Scale, Depression Anxiety Stress Scales Assessment, and Chinese Happiness Inventory	Decrease in stress in the AAT group more than in the control group
Anderson and Brown [24]; 2021	Stress and anxiety	Supportive	STAI^c^	Decreased anxiety in a convenience sample
Thakkar et al [25]; 2020	Stress and anxiety	Activating	Modified faces version of the Modified Child Dental Anxiety Scale	Effective behavior management strategy for children
Santaniello et al [26]; 2020	Neurological disorder	Therapeutic	MMSE^d^, GDS^e^	AAT showed an improvement in both cognitive function and mood
Brown et al [27]; 2019	Psychiatric disorder	Therapeutic	Wilcoxon signed rank test	Positive therapeutic impact on patients and staff in acute care psychiatric units, promoting positive mood and emotions
Hinic et al [28]; 2019	Stress and anxiety	Activating	STAI for Children	Reduce anxiety in hospitalized children and enhanced family satisfaction
Ginex et al [29]; 2018	Depression	Supportive	Patient Health Questionnaire–4	Promotes a healing environment for patients that involves a holistic and humanistic perspective
Priyanka MB [30]; 2018	Neurological disorder	Therapeutic	Autism Treatment Evaluation Checklist and semistructured interview for 15 min	Improved expression and communication skills when interacting with the dog, as well as noticeable enhancements in social and motor abilities
McCullough et al [31]; 2017	Stress and anxiety	Supportive	STAI	Help in reducing stress and anxiety levels
Branson at al [32]; 2017	Stress and anxiety	Supportive	STAI for Children, Positive and Negative Affect Schedule for Children	Increase positive feelings in hospitalized children
Nurenberg et al [5]; 2015	Psychiatric disorder	Therapeutic	Equine Assisted Growth and Learning Association, equine-assisted psychotherapy, canine-assisted psychotherapy	Effective therapeutic modality for long-term psychiatric patients at risk of violence
Stefanini et al [33]; 2015	Psychiatric disorder	Supportive	Children Global Assessment Scale	Significant improvements in overall functioning, a decrease in the need for specialized care, and an increase in regular school attendance
Menna et al [34]; 2015	Neurological disorder	Therapeutic	MMSE, 15-item GDS	Improved cognition and mood through repeated multimodal stimulation

a AAT: animal-assisted therapy.

b PTSD: posttraumatic stress disorder.

c STAI: State-Trait Anxiety Inventory.

d MMSE: Mini-Mental State Examination.

e GDS: Geriatric Depression Scale.

**Table 2.** Study characteristics.

Authors; year	Country	Study design	Patients					Study conditions	AAT^a^ description	Intervention	Control
			Number	Gender, n		Age (years), mean	Target group				
				Male	Female						
Shih and Yang [20]; 2023	Taiwan	Randomized controlled	90 (AAT group: 45; control group: 45)	45	45	50.2	Patients with schizophrenia	1-h therapy session once a week for 12 wk	Physical contact, brushing, playing, walking, and sitting	Dog-assisted therapy	Regular therapy
Chen et al [21]; 2022	Taiwan	Randomized controlled	40 (AAT group: 20; control group: 20)	18	22	54.6	Patients with schizophrenia	1-h therapy session once a week for 12 wk	15-min warm-up session like greetings, introduction, and season orientation; 45-min therapeutic activity, physical activities, or cognitive activities	Dog-assisted therapy	Regular therapy
Allen et al [22]; 2022	United States	Randomized controlled	33 (AAT group: 17; control: 16)	11	22	15	Abused youth with PTSD^b^	12 sessions, each lasting 90 min	Physical contact, petting	Dog present at the time of questionnaire	Dog was not present
Chen et al [23]; 2021	Taiwan	Randomized controlled	40 (AAT group: 20; control group: 20)	18	22	55.3	Patients with schizophrenia	1-h therapy session once a week for 12 wk	Petting, massaging, and playing with the dog (ball, loop, game)	Dog-assisted therapy	Regular therapy
Anderson and Brown [24]; 2021	United States	Randomized controlled	89 (AAT group: 45; control group: 44)	9	80	22.6	Nursing students anxiety	35-45 min before exam	Unstructured	Dog-assisted therapy	No interaction with dog
Thakkar et al [25]; 2020	India	Randomized controlled	100 (AAT group: 50; control group: 50)	44	56	7.5	Children who underwent dental assessment	Play with dog for 10-15 min in operatory	Petting and conversation with dog handler	Dog present during dental procedure	Dog was not present
Santaniello et al [26]; 2020	Italy	Randomized controlled	96 (AAT group- 65; control group-31)	23	75	75.8	Patients with Alzheimer disease	45-min therapy session once a week for 6 mo	Physical contact, brushing, playing, walking, and sitting	Dog present during therapy	Dog was not present during therapy
Brown et al [27]; 2019	United States	Time series and daily announcement	152 (adult inpatient unit: 84; adolescent inpatient unit: 68)	28; 18	56; 50	58	—^c^	Weekly dog visit	Interaction with therapy dog and its handler	Dog-assisted therapy	Regular therapy
Hinic et al [28]; 2019	United States	Purposive sampling	93 (AAT group: 50; control group: 43)	40	53	10.5	Hospitalized children	10-min pet therapy per visit	Dog present at the time of questionnaire; petting the dog	Dog-assisted therapy	No interaction with dog
Ginex et al [29]; 2018	United States	Randomized controlled	100 (AAT group: 50; control group: 50)	48	52	58	Patients of oncological surgical unit	4 d a week for 6 wk	Unstructured	Dog-assisted therapy	Regular therapy
Priyanka MB [30]; 2018	India	Purposive sampling	6	—	—	8.6	Children with autism	10 d for 12 wk	Petting and brushing the dog	Dog-assisted therapy	Regular therapy
McCullough et al [31]; 2017	United States	Randomized controlled	100 (AAT group: 60; control group: 46)	57	49	4.5	Patients with cancer	10-20 min per session	Petting and brushing the dog	Dog-assisted therapy	Regular therapy
Branson et al [32]; 2017	United States	Randomized controlled	48 (AAT group: 24; control group: 24)	24	24	13.4	Hospitalized children	10-min interaction in waiting room	Unstructured	Dog interaction	No interaction
Nurenberg et al [5]; 2015	United States	Randomized controlled	90	57	33	44.7	Chronic psychiatric inpatients	Weekly group session	Unstructured	Dog interaction	No interaction
Stefanini et al [33]; 2015	Italy	Randomized controlled	34 (AAT group: 17; control group: 17)	18	16	15	Acute mental patients	45-min therapy session once a week for 3 mo	Play activities, physical contact, grooming, cleaning	Dog interaction	No interaction
Menna et al [34]; 2015	Italy	Divided according to conditions	50 (AAT group: 40; control group: 10)	13	37	75.1	Patients with Alzheimer disease	45-min therapy session once a week for 6 mo	15-min reintroduction to dog; 20-min structured activity; 10-min same ending activity	Dog interaction	No interaction

a AAT: animal-assisted therapy.

b PTSD: posttraumatic stress disorder.

c Not available.

### Risk of Bias Determination

**Figure 2.**
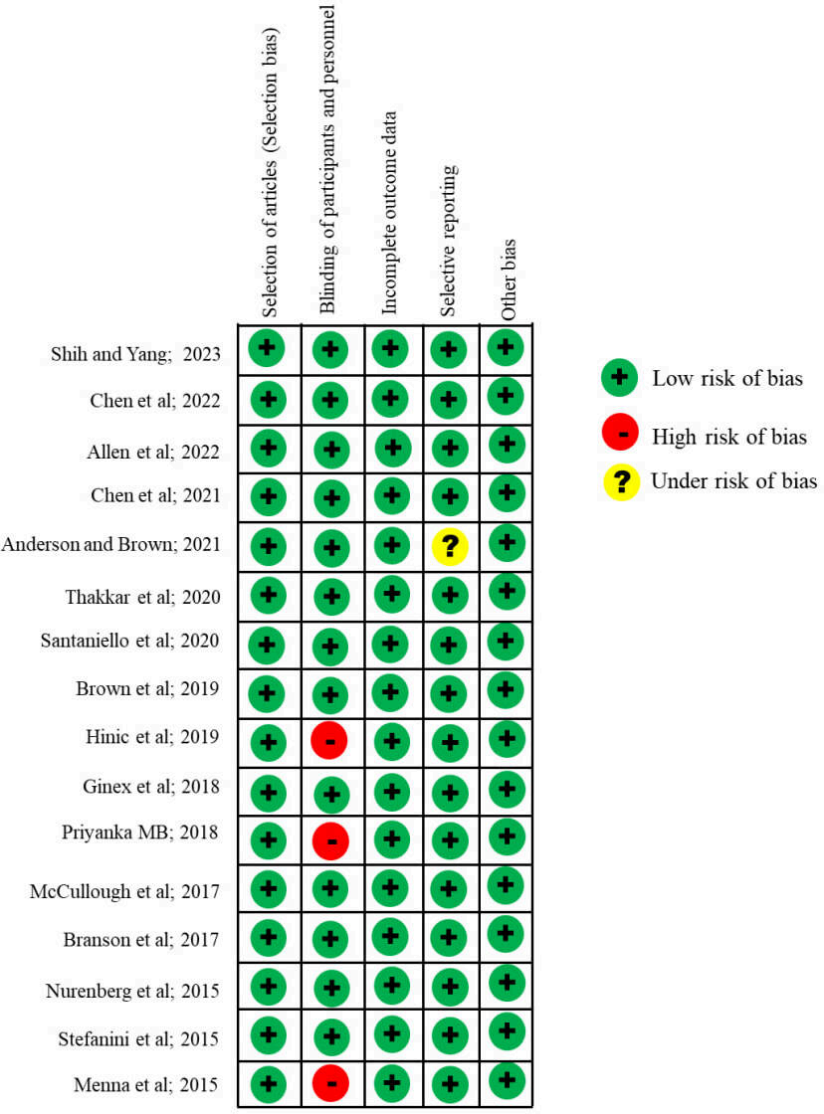
Risk of bias for the selected studies.

Figure 2 [5,20-34] provides a comprehensive evaluation of the overall risk and individual biases in each of the studies included. All the researchers carried out assessments to determine the likelihood of bias, and the results of these assessments showed a remarkable level of consistency across all the investigations. The data depicted in Figure 2 indicate that the articles authored by Hinic et al [28], Priyanka MB [30], and Menna et al [34] exhibited a high risk of bias. Additionally, the study conducted by Anderson and Brown [24] indicated a potential risk of bias. This consistent and rigorous approach enhances the confidence in the research paper’s results, underscoring the reliability of the reported biases and their impact on the study’s outcomes.

### General Characteristics

To provide a concise summary of the selected studies, we have compiled an overview in Table 2. It presents key information from each study, allowing for a quick and comprehensive understanding of the research landscape.

#### Quantitative Study

The studies analyzed in this research were conducted between 2015 and 2023, resulting in a total of 16 [5,20-34] included papers. These studies were carried out in various countries, including the United States (n=8), Taiwan (n=3), India (n=2), and Italy (n=3). Among the included studies, approximately 12 of 16 (75%) used a randomized controlled trial design. Two studies used conditional controlled designs, while 1 study followed a time series and daily announcement approach. The number of patients enrolled in the studies varied, ranging from 6 to 152 individuals. Specifically, 5 studies involved 0-40 patients, 2 studies included 41-80 patients, 8 studies comprised 81-120 patients, and 1 study encompassed 121-160 patients. The selected studies covered a diverse range of populations, with 7 studies focusing on children and adolescents from various disciplines including child psychology, psychiatry, and pediatrics. The same number of studies involved adult patients covering a range of fields such as general medicine, mental health, and geriatrics, and 2 studies specifically targeted older people, contributing to the fields of gerontology and geriatric medicine. In terms of gender distributions, women were more prominently represented, with ≥50% female participants in 13 of the 16 studies (Table 2).

The interventions included in the studies were described using various terms such as pet encounter therapy, pet-facilitated therapy, pet-assisted living, animal-assisted intervention, AAT, animal-assisted activity, or simply dog visits/therapy. All of the studies incorporated dogs as the primary intervention. In terms of the duration of the interventions, the 7 studies had varying time periods per visit [5,24,25,27,28,31,32]. Five studies had interventions lasting for 12 weeks [20,21,23,30,33] and one for 6 weeks [29], while 2 studies had longer intervention periods of 6 months [26,34]. One study did not explicitly mention the duration of the intervention [22]. The majority of the studies used a one-to-one approach in delivering the intervention, emphasizing individual interactions between participants and the dogs. However, 1 study was conducted in a group setting [5]. Most studies actively encouraged touching and interaction with the animals, while in 2 studies, the interaction was described as unstructured [24,32].

#### Qualitative Study

The description of selected studies for qualitative analysis is presented in Tables1  2. Studies reported on patients’ experiences with animal-assisted interventions such as dog therapy or animal visits. Thematic analysis was used to identify recurring themes and extract meaningful insights from the type of disorder. Participants described the animals as a source of comfort, providing emotional support and reducing stress and anxiety. The interactions with the animals were reported to have a soothing effect and helped individuals cope with their challenges and emotional difficulties.

The qualitative analysis shed light on the subjective experiences and perceptions of individuals participating in the interventions. It provided valuable insights into the emotional, social, and therapeutic benefits associated with animal-assisted interventions, highlighting the potential of these interventions to enhance well-being and quality of life. The selected studies on psychiatric disorders predominantly focused on schizophrenia, with 5 studies specifically addressing this condition in adults. Additionally, 1 study explored acute mental disorders in children [33]. Six studies were dedicated to investigating stress and anxiety, targeting various populations such as children undergoing physical examinations, children with PTSD, patients with cancer, and nursing students. Three studies examined neurological disorders, including 1 study involving children with autism [30] and 2 studies involving older individuals with Alzheimer disease [26,34]. In 1 study, the intervention aimed to reduce depression among patients undergoing oncology surgery [25].

#### Outcomes

The number of studies with at least one statistically significant positive outcome measure, divided by patient condition and intervention category, is presented in Table 3. The study aimed to comprehensively evaluate mental health, social functioning, and overall quality of life, taking into account various parameters specific to each measurement scale, for example, generic health-related quality of life measures like Posttraumatic Stress Disorder Reaction Index for DSM-5, State-Trait Anxiety Inventory (STAI) for Children, Patient Health Questionnaire–4 (PHQ-4), Mini-Mental State Examination (MMSE), and 15-item Geriatric Depression Scale (GDS), and general functional measures such as Mental Health–Social Functioning Scale, Social Adaptive Function Scale, chair stand test, Timed Up and Go, Assessment of Communication and Interaction Skills, etc.

**Table 3.** Number of studies classified based on the condition, type of intervention, and the presence of positive outcome.

Condition	Supportive mediation, n		Therapeutic mediation, n		Activating mediation, n	
	Yes	No	Yes	No	Yes	No
Psychiatric disorder	1	0	4	1	0	0
Neurological disorder	0	0	3	0	0	0
Stress and anxiety	3	0	0	0	2	1
Depression	1	0	0	0	0	0

#### Psychiatric Disorder

All 6 trials that focused on psychiatric disorders were categorized as AAT and involved interventions with dog therapy. Among these studies, 5 were conducted using a randomized controlled design, while 1 study used a time series design with randomized daily announcements within a pre-post experimental framework. One study specifically examined patients in child and adolescent psychiatry [33], while the remaining 5 studies focused on adult psychiatry patients [5,20,21,23,27]. The duration of the AAT programs varied, with some studies consisting of 12-week programs in different settings, while 2 studies provided weekly therapy sessions without specifying the intervention period [5,27].

Each of the 6 conducted studies involved a comparison between an intervention group receiving a specific therapy and a control group that did not participate in any related activities. Notably, the 5 studies specifically targeted middle-aged and older patients diagnosed with chronic schizophrenia. The results of these studies consistently demonstrated significant improvements in various areas, including reductions in psychiatric symptoms, enhanced social functioning, improved quality of life, enhanced cognitive function, increased agility and mobility, and decreased stress levels. These outcomes were measured using a variety of scales and assessment tools [5,20,21,23,33]. In a study conducted by Brown et al [27], the focus was on examining the impact of mood states and feelings among patients and staff in inpatient psychiatric units. The researchers observed significant changes in mood before and after sessions involving therapy dogs. Specifically, negative moods decreased, while positive moods, such as feelings of happiness, relaxation, and calmness, increased. These changes were measured using the visual analog mood scale [27]. Overall, these findings highlight the efficacy of AAT in positively impacting the well-being and overall functioning of individuals with psychiatric disorders.

#### Neurological Disorder

Among the studies that focused on neurological disorders, 3 used dog therapy as an intervention. One of these studies used a randomized controlled design [26], while the other 2 studies used purposive sampling based on the patients’ conditions. One study specifically targeted children and adolescents with autism, while the other 2 studies focused on older patients with Alzheimer disease. The duration of the AAT programs varied, ranging from 3 to 6 months.

In each of the 3 conducted studies, the intervention group was compared to a control group that did not participate in any activities to assess the outcomes of the therapy. Priyanka MB’s [30] study focused on children with autism and observed that engaging with a therapy dog, such as brushing the dog and attempting to draw and write for the dog, led to enhanced social and motor skills. Additionally, the children experienced a sense of relaxation and calmness in the presence of the dog. The studies conducted by Menna et al [34] and Santaniello et al [26], focusing on older patients with Alzheimer disease over 6 months, have shown promising results. Menna et al’s [34] study demonstrated the applicability and effectiveness of AAT interventions in stimulating cognition and improving mood. The interventions involved repeated multimodal stimulation, including verbal, visual, and tactile approaches. Similarly, Santaniello et al’s [26] study also revealed improvements in both cognitive function and mood in the AAT group, as measured by changes in the MMSE and GDS. Overall, these studies indicate that nonpharmacological therapies, particularly AAT, have the potential to reduce symptoms associated with neurological disorders.

#### Stress and Anxiety

The 6 trials that specifically addressed stress and anxiety used AAT interventions involving dog therapy. These studies exclusively targeted children and adolescents, using a randomized controlled design. In 5 of the studies, the therapy sessions lasted between 10-45 minutes, while 1 study did not specify the duration of the intervention period.

The study conducted by Allen et al [22] focused on youths who had experienced abuse and were diagnosed with PTSD. The results revealed that the group receiving the intervention showed greater improvements in caregiver-reported symptoms of PTSD, internalizing concerns, and externalizing problems compared to the control group [22]. In a study by Anderson and Brown [24] involving nursing students, the intervention group experienced interactions with dogs before testing. This interaction served as a stress reliever for the students, resulting in a decrease in anxiety as measured by the STAI. Thakkar et al [25] conducted a study on children who were undergoing dental assessments. The findings indicated that the intervention group showed a significantly greater anxiety reduction compared to the control group, as measured by the modified faces version of the Modified Child Dental Anxiety Scale. In the studies conducted by Hinic et al [28] and Branson et al [32], dog therapy was provided to children who were hospitalized, and their anxiety levels were assessed before and after the intervention. The results from the STAI for Children suggested that brief pet therapy visits served as a tool to decrease anxiety in children who were hospitalized and promote family satisfaction. McCullough et al [31] conducted a study where the intervention group participated in dog therapy, while the control group received standard care at the hospital. The findings demonstrated the applicability and effectiveness of AAT interventions in reducing stress and anxiety levels in patients with cancer.

Overall, when considering the results of all these studies, it becomes evident that each one exhibited at least one statistically significant positive effect. When these findings are examined collectively, they provide compelling evidence to suggest that particular modalities of AAT hold substantial promise in terms of reducing stress levels and fostering a positive impact on individuals’ overall mood and well-being.

#### Depression

Ginex et al [29] conducted a study to explore the impact of a dog-assisted intervention on an inpatient surgical oncology unit. The study used a randomized controlled design, with patients in the intervention group receiving therapy 4 days per week throughout the study period. In contrast, the control group underwent physical therapy without any modifications to their normal routine. Patients in the intervention group reported a significant decrease in depression and anxiety levels, as measured by the PHQ-4, compared to the control group. The findings of the study suggest that AAT fosters a healing environment for patients, incorporating a holistic and humanistic approach that elicits overwhelmingly positive responses.

## Discussion

The outcomes of this meta-analysis provide the long-standing belief that animals can play a beneficial role in the healing process. The study revealed positive and moderately strong results across various aspects, including medical well-being, behavioral outcomes, and the reduction of autism spectrum symptoms. Moreover, the effect on all four outcomes, which include psychiatric disorders, neurological disorders, stress and anxiety, and depression, were consistent and uniform. Additionally, support for AAT was evident from 4 studies comparing it with established interventions, demonstrating that AAT was equally or more effective. These compelling findings indicate that AAT is a robust intervention deserving of further exploration and use. This systematic review and meta-analysis specifically focused on dogs as the assisting animals in a health care setting. However, there were no limitations on the characteristics of the population included in the study. Although this research synthesis provides evidence in favor of the effectiveness of AAT, it is essential to acknowledge the complexities associated with interventions in general and the specific nuances related to the use of AAT.

The majority of articles included in this systematic review were based on randomized controlled trials conducted in various countries. Additionally, time series and daily announcements, divided according to different conditions, were also considered. The increased number of studies provided greater support in assessing the variance of heterogeneity and potential group differences. Although the results are speculative, the meta-analysis demonstrated homogeneity in the summary values, with only one exploratory group difference reaching statistical significance. Nonetheless, this analysis brought forth several intriguing questions and patterns, serving as a foundation for discussions or further research on the factors influencing the effectiveness of AAT. For instance, consistent benefits were observed in children, young age groups, and old age groups across all outcome variables, including symptoms associated with psychiatric disorders, stress, and anxiety [35]. In particular, among the adult group, a high prevalence of psychiatric disorders, followed by neurological disorders, stress, and anxiety, was found. In contrast, in children, a high number of cases related to stress and anxiety disorders were identified.

Several organizations in different countries are actively working to promote AAT. At the global level, the International Association of Human-Animal Interaction is the worldwide consortium of organizations involved in the practice, research, or education of AAT and the training of service animals [36]. In the United States, the Society for Healthcare Epidemiology of America has established comprehensive guidelines for animals in health care facilities, which emphasize the importance of written policies, designated AAI visit liaisons, and formal training programs for animals and handlers [37]. However, despite these guidelines, there is no legal requirement for health care facilities to adopt these measures. One notable organization in the United States, Pet Partners, stands out as the only national therapy animal organization that mandates volunteer training and biennial evaluations of animal-handler teams, and prohibits raw meat diets [38]. In Europe, the European Society for Animal Assisted Therapy (ESAAT) plays a substantial role as an influential organization operating across various disciplines and professions within the field. ESAAT’s primary mission is to accredit education and training programs in the domain of animal-assisted interventions [39]. While the Western world has made significant advancements in AAT, Eastern countries such as India, China, Taiwan, Japan, and Sri Lanka are still in the early stages of exploring and implementing such practices. These countries are currently in the infancy phase of using and developing their own AAT programs. As awareness and understanding of the benefits of AAT continue to grow worldwide, it is expected that these Eastern countries will gradually catch up and further enhance their ATT initiatives [40].

Our review was based on a limited number of studies, which can be attributed to our strict inclusion criteria and the presence of suboptimal study designs. Specifically, many of the randomized trials were characterized by small sample sizes, short durations, and a lack of follow-up assessments. Another limitation pertains to the suitability of the outcome measures used, which may not fully capture the important values and impacts as perceived by the participants. On the other hand, the qualitative research included in the review exhibited higher overall quality and contributed valuable insights to our findings.

In conclusion, the reviewed studies provide preliminary evidence of the potential benefits of AAT in certain conditions. It suggests that dog-assisted therapy can have minor to moderate effects in treating psychiatric disorders, cognitive disorders, neurological disorders, etc, and demonstrates potential in various medical interventions. However, it is important to note that some of the outcome measures analyzed did not show significant effects, and further research is needed to better understand the specific contexts and conditions. To foster the growth of such therapy, we need education campaigns, research programs, professional support, and media awareness to increase the effectiveness of AAT across different countries.

## Supplementary material

10.2196/51787 Checklist 1 PRISMA (Preferred Reporting Items for Systematic Reviews and Meta-Analyses) checklist

## Abbreviations

AAT: animal-assisted therapy
ESAAT: European Society for Animal Assisted Therapy
GDS: Geriatric Depression Scale
MeSH: Medical Subject Headings
MMSE: Mini-Mental State Examination
PHQ-4: Patient Health Questionnaire–4
PICOS: Patient, Problem, or Population; Intervention; Comparison; Outcomes; and Study Design
PRISMA: Preferred Reporting Items for Systematic Reviews and Meta-Analyses
PTSD: posttraumatic stress disorder
STAI: State-Trait Anxiety Inventory
